# Rhinovirus/enterovirus contribution to respiratory-associated hospitalizations in adults during respiratory seasons in Spain: A 6-year prospective study

**DOI:** 10.1371/journal.pone.0347659

**Published:** 2026-04-20

**Authors:** Sandra S. Chaves, Valérie Bosch Castells, Ainara Mira-Iglesias, Joan Puig-Barberà, F. Xavier López-Labrador, Miguel Tortajada-Girbés, Mario Carballido-Fernández, Joan Mollar-Maseres, Germán Schwarz-Chávarri, Javier Díez-Domingo, Alejandro Orrico-Sánchez, Valencia Hospital Network for the Study of Influenza and other Respiratory Viruses (VAHNSI)

**Affiliations:** 1 Sanofi Vaccines, Lyon, France; 2 Vaccine Research Department, Fisabio – Public Health, Valencia, Spain; 3 CIBER-ESP, Instituto de Salud Carlos III, Madrid, Spain; 4 Virology Laboratory, Fisabio – Public Health, Valencia, Spain; 5 Department of Microbiology and Ecology, Medical School, University of Valencia, Valencia, Spain; 6 Hospital Universitario Doctor Peset, Valencia, Spain; 7 Hospital General Universitario de Castellón, Castellón, Spain; 8 Medicine Department, Universidad CEU Cardenal Herrera, Castellón, Spain; 9 Hospital Universitario y Politécnico La Fe, Valencia, Spain; 10 Hospital General Universitario Dr. Balmis, Alicante, Spain; 11 Universidad Católica de Valencia San Vicente Mártir, Valencia, Spain; Università degli Studi di Parma: Universita degli Studi di Parma, ITALY

## Abstract

**Background:**

Understanding the burden of acute viral respiratory infection-related hospitalizations is crucial for guiding research and development. Unlike influenza, respiratory syncytial virus (RSV), or severe acute respiratory syndrome coronavirus 2, no pharmaceutical interventions exist for other respiratory viruses; therefore, their impact remains poorly characterized. This study aimed to investigate the association of current non-vaccine-preventable respiratory viruses, especially rhinovirus/enterovirus (RV/EV), on hospitalizations during the respiratory seasons.

**Methods:**

Data from a prospective study that used multiplex polymerase chain reaction to conduct long-term surveillance on respiratory viruses in Valencia, Spain were analyzed. Patients aged ≥50 years hospitalized due to respiratory illness from 2014–15–2019–20 were included.

**Results:**

Respiratory viruses were detected in 35.2% (3,755/10,675) of hospitalized patients with acute respiratory illness. Influenza and RSV accounted for 22.1% of hospitalizations, RV/EV for 7.6%, and other non-vaccine-preventable viruses for 5.4%. Adults ≥75 years had average seasonal hospitalization incidence rates more than twice those aged 65–74 years and eight times those aged 50–64-year-olds. No significant differences in severity markers were observed among patients with or without virus identified, those aged ≥75 years had a 2–3 times higher mortality rate compared to younger age groups.

**Conclusions:**

The potential impact of respiratory viruses on hospitalization rates among older adults, particularly those aged ≥75 years, highlights the need for targeted interventions to reduce healthcare system burden. Enhanced diagnostic capabilities and the development of next-generation preventive strategies, including vaccines and therapeutics, could improve patient outcomes and strengthen the resilience of the healthcare system during respiratory virus seasons.

## Introduction

Lower respiratory tract infections are a leading cause of morbidity and mortality globally, affecting the very young and the elderly disproportionately [1]. In Europe, respiratory system diseases are the third most common cause of death [2], contributing significantly to the public health burden. Among respiratory pathogens, influenza virus, respiratory syncytial virus (RSV), and severe acute respiratory syndrome coronavirus 2 (SARS‑CoV‑2) are major contributors to severe illness and mortality among adults with underlying chronic medical conditions and those 65 years and older [1,3–5]. There are relatively robust surveillance systems to monitor these viruses; however, other respiratory viruses, such as parainfluenza virus, human metapneumovirus (HMPV), and human rhinovirus (RV), remain comparatively understudied in these populations, despite mounting evidence of their role in infections leading to hospitalizations [6–9].

In temperate regions, especially during winter months, all these viruses co-circulate, adding pressure to respiratory illness associated healthcare utilization [10,11].Vaccines and therapies for influenza [12], RSV [5], and SARS-CoV-2 [13] are currently available. However, there are no pharmaceutical interventions for other respiratory viruses. Hence, their clinical and epidemiological impact remains inadequately characterized, creating a significant knowledge gap in respiratory disease management.

To improve our understanding of the contribution of other respiratory viruses to hospitalizations among adults, we analyzed data from a prospective study examining patients aged ≥50 years hospitalized because of respiratory illness. We used comprehensive viral panel testing to investigate the relative impact of different respiratory viruses on hospitalization incidence rates and disease severity during the respiratory season, while focusing on characterizing the impact of RV infections separately. However, because of the genetic similarities between enterovirus (EV) and RV, the molecular methods we used may have misclassified some EV as RV, as available assays based on the highly conserved 5′ non-coding region may not distinguish between these viruses for an accurate diagnosis [14]. Therefore, we reported laboratory results as combined RV/EV positive results for our analysis, where the associated hospitalizations occurred in the respiratory season, where RV circulation peaks in the fall months [15,16].

## Materials and methods

### Study setting and design

We analyzed hospital‐based active-surveillance data obtained from the Valencia Hospital Surveillance Network for the Study of Influenza and Other Respiratory Viruses (VAHNSI) in Valencia, Spain. VAHNSI conducted prospective active surveillance on patients hospitalized because of acute respiratory illness from November 1 through March 31 (respiratory season). Although the surveillance was conducted on patients of all ages, our analysis focused on patients aged ≥50 years who were hospitalized during the respiratory seasons, from 2014–15–2019–20. The 2019–20 season was interrupted on March 14^th^, when the government approved the declaration of a state of emergency throughout Spain to deal with the health emergency caused by SARS-CoV-2. Ten hospitals provided data in the 2014–15 season and four hospitals during the other seasons. The catchment area of these hospitals covered 21% to 46% of inhabitants in Valencia Region (approximately 5 million) of Spain. The study methodology has been previously described [17].

### Case definition and enrollment

Residents in the catchment area of one of the participating hospitals who were non-institutionalized and had not been discharged from a previous admission in the past 30 days were eligible to be enrolled if they were hospitalized because of acute respiratory illness, which was defined as at least one respiratory (cough, sore throat, or shortness of breath) and one systemic (fever or feverishness, headache, myalgia, or malaise) sign/symptom with an onset of ≤7 days [18]. Detailed clinical and demographic data were gathered through patient interviews and medical chart abstraction after obtaining informed consent from patients.

### Data collection

The demographic data collected for this study included sex, age at the day of admission, smoking status, body mass index (BMI), and functional dependency (measured by the Barthel index for patients aged ≥65 years). Clinical data collected included the length of hospital stay (LOS), the presence of chronic medical conditions, admission to the intensive care unit (ICU), the use of mechanical ventilation, and the date of discharge or death. Chronic medical conditions included heart disease, cerebrovascular disease, peripheral arteriopathy, asthma, lung disease, diabetes, endocrine system disease other than diabetes, anemia, chronic liver disease, chronic renal disease, chronic autoimmune disease, neurological/neuromuscular diseases, and neoplastic disease.

### Laboratory methods

Nasopharyngeal and/or oropharyngeal swabs were obtained from each patient. Swabs were placed in a single 3-mL Universal Transport Medium tube (Copan, Italy) and stored at ≤−20 °C at the study site or dispatched refrigerated directly to the reference laboratory of the coordinating site for testing. Samples were collected within 48 h of hospital admission and tested in batches of 22 at a centralized laboratory (FISABIO-Public Health, Valencia, Spain). One-third of the viral transport medium volume (1 mL) was used for the extraction of total nucleic acids using an automated silica-based method (Nuclisens Easy-Mag, BioMérieux, Lyon, France). Subsequently, the extracted nucleic acids were analyzed using a real-time reverse transcription polymerase chain reaction (RT-PCR) multiplex panel to test for the presence of adenovirus, bocavirus, seasonal human coronaviruses (229E, HKU1, NL63, and OC43), HMPV, parainfluenza viruses [1–4], RSV (A/B), RV (A/B/C), and influenza (A/B), as described previously [17], using Roche LightCycler^®^ 480 II with some modifications. An additional RV primer was included for enhanced detection of RV-C species [19], the master mix used from 2015 onward was qScript XLT 1-Step RT-qPCR ToughMix (Quantabio, MA, USA), and probes for RSV-B were updated in 2017 [20]. Because the genetic similarities between EV and RV, the results are presented as EV/RV to account for cross-reaction among these viruses.

### Statistical analyses

For this analysis, we categorized patients into age groups 50–64 years, 65–74 years, and ≥75 years. The LOS was recorded as the number of nights spent in the hospital. Admission to the ICU, need for mechanical ventilation, LOS, and deaths were considered markers of disease severity, and severe cases were defined as patients who were admitted to the ICU, required mechanical ventilation, or died during hospitalization (as a composite variable). We also grouped the viral results into the following categories: vaccine-preventable (i.e., influenza viruses and RSV), RV/EV, and other non-vaccine-preventable viruses (i.e., HMPV, parainfluenza viruses, adenovirus, seasonal human coronaviruses, and bocaviruses). We excluded all cases with viral co-detections to report only the impact of individual virus on hospitalizations.

Categorical data were described as proportions and compared using Chi-square tests, or Fisher’s exact tests as appropriate. Continuous data were compared using Student’s *t*-tests, or Wilcoxon tests when the conditions for applying a student’s *t*-test were not met. A p-value <0.05 indicated statistical significance. Incidence rates were calculated as the number of hospitalizations divided by the population of the VAHNSI catchment area stratified by age group and expressed per 100,000 individuals during each defined season. We estimated the mean incidence rates by hospitalization category and patient age, and then, presented the mean incidence (observed across the seasons) and the maximum and minimum rates for specific seasons. All analyses were conducted using R software.

### Ethics declaration

The Ethics Research Committee of the Dirección General de Salud Pública-Centro Superior de Investigación en Salud Pública (DGSP-CSISP) approved the original protocol of the study. Patients included in this analysis were enrolled from November 10^th^ 2014 through March 14^th^ 2020, providing written informed consent before their inclusion. Additional information regarding the ethical, cultural, and scientific considerations specific to inclusivity in global research is included in the Supporting Information.

## Results

### Demographic characteristics and underlying medical conditions

From 2014–15–2019–20, 10,833 patients aged ≥50 years were hospitalized because of acute respiratory illnesses. A total of 64 samples that were damaged, lost, or yielded inconclusive results and an additional 94 cases with viral co-detections were excluded. We included 10,675 patients in this analysis. Of these patients, 60.4% were ≥75 years old, 53.4% were male, 47% had never smoked, 31.8% had a normal BMI, and 56.7% had minimal functional dependency. The majority (89.6%) of hospitalized patients had at least one underlying chronic disease, the most common being heart disease (50.9%), followed by lung disease (36.0%) and diabetes (33.3%). Almost one-third of hospitalizations (29.5%) were registered during the 2014–15 season (wherein 10 hospitals participated in the surveillance), whereas <10% occurred during the 2019–20 season, which was impacted by the emergence of SARS-CoV-2 (Table 1).

**Table 1:** Demographic characteristics of patients aged ≥50 years hospitalized because of acute respiratory illness, by the presence of single respiratory viruses detected or no virus detected. Valencia, Spain, 2014–2020.

	All acute respiratory-associated hospitalizations^d^ (N = 10,675)		Hospitalizations associated with single virus detected (n = 3,755)		Hospitalizations with no virus detected (n = 6,920)	
	n	%	n	%	n	%
**Age (years)**						
50–64	1788	16.7	575	15.3	1213	17.5
65–74	2434	22.8	863	23.0	1571	22.7
≥75	6453	60.4	2317	61.7	4136	59.8
**Sex**						
Male	5704	53.4	1870	49.8	3834	55.4
Female	4971	46.6	1885	50.2	3086	44.6
Ratio M/F	1.1		1		1.2	
**Smoking status**						
Never	5015	47.0	1888	50.3	3127	45.2
Current	1427	13.4	498	13.3	929	13.4
Former	4228	39.6	1368	36.4	2860	41.3
**BMI** ^**a**^						
Underweight	216	2.0	84	2.2	132	1.9
Normal	3392	31.8	1138	30.3	2254	32.6
Overweight	3956	37.1	1427	38.0	2529	36.5
Obese	2809	26.3	1002	26.7	1807	26.1
Morbid obese	302	2.8	104	2.8	198	2.9
**Functional dependency** ^**b**^						
Total	872	9.8	287	9.0	585	10.3
Severe	1041	11.7	343	10.8	698	12.2
Moderate	1559	17.5	580	18.2	979	17.2
Mild	382	4.3	150	4.7	232	4.1
Minimal	5035	56.7	1820	57.2	3215	56.3
**Number of comorbidities at admission**						
None	1106	10.4	362	9.6	744	10.8
At least one	9569	89.6	3393	90.4	6176	89.2
Two or more	6750	63.2	2394	63.8	4356	62.9
**Comorbidity** ^**c**^						
Heart disease	5429	50.9	1914	51.0	3515	50.8
Cerebrovascular disease	680	6.4	241	6.4	439	6.3
Peripheral arteriopathy	473	4.4	156	4.2	317	4.6
Asthma	924	8.7	354	9.4	570	8.2
Lung disease	3838	36.0	1346	35.8	2492	36.0
Diabetes	3552	33.3	1220	32.5	2332	33.7
Endocrine system disease other than diabetes	1270	11.9	523	13.9	747	10.8
Anemia	1151	10.8	407	10.8	744	10.8
Chronic liver disease	449	4.2	139	3.7	310	4.5
Chronic renal disease	1859	17.4	666	17.7	1193	17.2
Chronic autoimmune disease^d^	504	4.7	216	5.8	288	4.2
Neurological/ neuromuscular diseases ^e^	1185	11.1	379	10.1	806	11.6
Neoplastic disease	1015	9.5	341	9.1	674	9.7
**Season** ^**f**^						
2014–15	3145	29.5	1114	29.7	2031	29.3
2015–16	1566	14.7	481	12.8	1085	15.7
2016–17	1415	13.3	474	12.6	941	13.6
2017–18	1690	15.8	784	20.9	906	13.1
2018–19	1807	16.9	655	17.4	1152	16.6
2019–20	1052	9.9	247	6.6	805	11.6

^a^The Body mass index (BMI) categories were defined by underweight (<18.5), normal (18.5 to <25), overweight (25 to <30), obese (30 to <40), and morbid obese (≥40).

^b^The Barthel Index data were only available for only patients aged ≥65 years.

^c^The rows don’t add up to 100% as comorbidities are were not mutually exclusive.

^d^Includes acquired or hereditary immunodeficiencies and chronic autoimmune disease (e.g., lupus, or rheumatoid arthritis).

^e^Includes neuromuscular or neurodegenerative disease and senile dementia or Alzheimer’s disease.

^f^Ten hospitals contributed to the season 2014–2015, while whereas four hospitals participated in other seasons.

Abbreviations: BMI, body mass index; F, female; M, male.

The characteristics of hospitalized patients identified at admission were similar among those with or without respiratory virus detection. The percentage of hospitalizations associated with the 2017–18 season in the group with respiratory viruses (20.9%) was higher than that in those without detectable respiratory viruses (13.1%) (Table 1). Patients’ demographic characteristics were similar when compared by season (S1 Table).

### Time-dependent variation on virus detection

**Fig 1:**
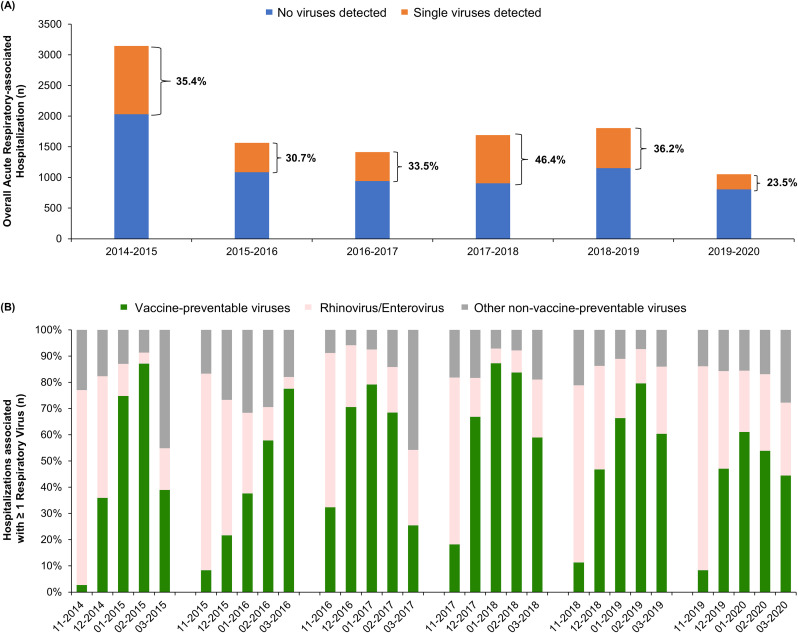
Contribution of respiratory viruses to overall acute respiratory-associated hospitalizations during the respiratory seasons among adults aged ≥50 years in Valencia, Spain, 2014-2020. (A) shows the total number of hospitalizations by season and the contribution of single virus detection, indicated in percentage. (B) shows the monthly distribution of vaccine-preventable viruses (influenza and RSV), rhinovirus/enterovirus and other non-vaccine-preventable viruses (parainfluenza viruses, adenovirus, seasonal coronavirus, HMPV, and bocavirus) to the volume of hospitalizations among those with single virus detected by season.

A virus was detected in specimens from 3,755 (35.2%) patients, with seasonal variation in their contribution to acute respiratory hospitalizations—ranging from 23.5% in 2019–20 to 46.4% in 2017–18 (Fig 1A). The monthly contribution of different viruses showed significant patterns: vaccine-preventable viruses (influenza and RSV) accounted for 10% to 85% of viral hospitalizations, depending on the month and season. RV/EV-associated hospitalizations dominated the early respiratory season (November to December), whereas vaccine-preventable virus hospitalizations typically peaked between January and February, except for the 2015–16 season that showed a delayed peak in March (Fig 1B).

### Severity of hospitalization by virus type

The median LOS among all hospitalized patients with respiratory illness was 6 days (interquartile range: 4–8 days), with 2.0% of them admitted to the ICU and 5.3% requiring mechanical ventilation, and 5.1% died while hospitalized. Hospitalizations were characterized as severe for 10.8% of patients. Univariate analysis showed that severity markers did not differ whether respiratory viruses were detected or not or whether the hospitalization was associated with vaccine-preventable viruses (Table 2).

**Table 2:** Severity indicators of hospitalizations with and without respiratory virus detected at admission and whether the virus detected were vaccine preventable or not, with distribution by age group. Valencia, Spain 2014–2020.

	N	Length of stay, in days (median [IQR])	ICU		Mechanical ventilation^b^		Death		Severe case^c^	
			n	%	n	%	n	%	n	%
**All acute respiratory associated hospitalizations**	10675	6 (4; 8)	210	2.0	564	5.3	525	4.9	1,133	10.6
**No virus detected**	6920	6 (4; 8)	146	2.1	367	5.3	337	4.9	741	10.7
**Single virus detected**	3755	6 (4; 8)	64	1.7	197	5.2	188	5.0	392	10.4
**Vaccine-preventable viruses** ^**a**^	2361	6 (4; 8)	47	2.0	136	5.8	120	5.1	260	11.0
50 to 64 years (n = 346)		6 (4;9)	14	4.0	25	7.2	4	1.2	32	9.2
65-74 years (n = 547)		5 (4;8)	12	2.2	36	6.6	18	3.3	55	10.1
≥75 years (n = 1468)		6 (4;8)	21	1.4	75	5.1	98	6.7	173	11.8
**RV/ EV**	815	6 (4; 8.75)	9	1.1	39	4.8	44	5.4	83	10.2
50 to 64 years (n = 151)		6 (4;8)	2	1.3	11	7.3	3	2	14	9.3
65-74 years (n = 181)		6 (4;9)	2	1.1	12	6.6	4	2.2	17	9.4
≥75 years (n = 483)		6 (4;9)	5	1.0	16	3.3	37	7.7	52	10.8
**Other non-vaccine preventable viruses** ^**a**^	579	6 (4; 8)	8	1.4	22	3.8	24	4.1	49	8.5
50 to 64 years (n = 78)		6 (4;8)	3	3.8	6	7.7	0	0.0	8	10.3
65-74 years (n = 135)		6 (4;8)	1	0.7	4	3.0	2	1.5	7	5.2
≥75 years (n = 366)		5,5 (4;8)	4	1.1	12	3.3	22	6.0	34	9.3

^a^Vaccine-preventable viruses encompassed influenza viruses and RSV; non-vaccine-preventable viruses were defined as RV/EV, HMPV, parainfluenza viruses, seasonal human coronaviruses, adenovirus, and bocavirus. Co-detections were removed from this analysis.

^b^Twelve patients requiring extracorporeal membrane oxygenation were combined with those requiring mechanical ventilation for the analysis.

^c^The composite variable was defined as a patient admitted to the ICU, who required mechanical ventilation, or died during hospital stay.

NOTE: All comparison of death distribution by age groups were statistically significant different (p < 0.001). Those aged 50–64 years in the group of vaccine-preventable viruses were significantly more likely to be admitted to the ICU (p < 0.01), no other comparisons were statistically significant.

HMPV, human metapneumovirus; ICU, intensive care unit; IQR, interquartile range; RSV, respiratory syncytial virus.

The analysis by age group showed that mortality rate was two to three times higher among those aged ≥75 years than that among their younger counterparts, and the difference was significant whether the virus detected was vaccine-reventable or not. There were significantly more ICU admissions among patients aged 50–64 years in the group of vaccine-preventable viruses. The number of patients admitted to the ICU was small for the other categories.

### Incidence rate

**Fig 2:**
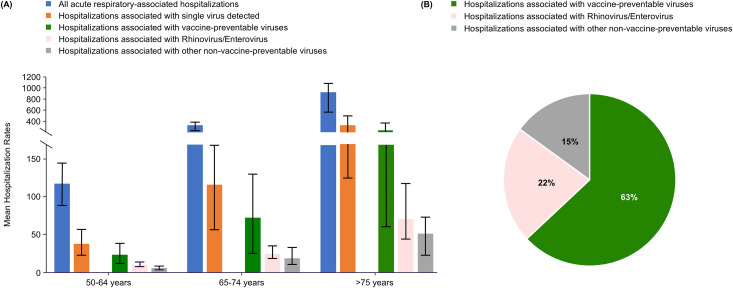
Acute respiratory-associated hospitalizations by age group and potential viral etiology (A) shows mean annual incidence of hospitalizations per 100,000 individuals associated with acute respiratory hospitalizations during the respiratory seasons, with and without virus detected at admission, and, among those with single virus detected, the distribution of vaccine-preventable and non- vaccine-preventable respiratory viruses, by age group in Valencia, Spain, 2014–2020. (B) shows the percentage distribution of the respiratory viruses (vaccine preventable, rhinovirus/enterovirus, and other non-vaccine preventable) to overall mean hospitalization incidence rates for viral associated hospitalizations.

Among adult population aged ≥50 years, the seasonal mean rate of acute respiratory illness hospitalizations was 357.5 per 100,000 population, ranging from 234.4 to 410.4 per 100,000 population during the study period. Of these, hospitalizations with a single virus detected at admission averaged 125.8 per 100,000 population (S2 Table; Fig 2A). Vaccine-preventable viruses were responsible for 63% of the cases (78.8 per 100,000 population), whereas RV/EV accounted for 22% (27.8 per 100,000 individuals) (Fig 2B). Those aged ≥75 years were disproportionally represented, with the overall average seasonal hospitalization rate of 925.9 per 100,000 individuals compared with that of 328.9 per 100,000 individuals among those aged 65–74 years and 118.1 per 100,000 individuals among those aged 50–64 years (S2 Table; Fig 2A). A similar distribution of hospitalization rates by age group was observed regardless of whether the detected virus was vaccine-preventable or not, meaning that the hospitalization rates for those aged ≥75 years were nearly three times higher than those aged 65–74 years and seven to nine times higher than those aged 50–64 years.

## Discussion

Our findings demonstrate that respiratory viruses were detected in approximately one-third of all acute respiratory-associated hospitalizations in adults aged ≥50 years during the respiratory season (November through March) from 2014 to 2020, varying from ~20% to 46% depending on the season. Although influenza and RSV—both vaccine-preventable—accounted for most virus-associated hospitalizations, other respiratory viruses have also potentially impacted overall hospitalization rates. Notably, RV/EV were detected in 22% of viral respiratory hospitalizations, surpassing the combined contribution of all other non-vaccine-preventable viruses. Adults aged ≥75 years were disproportionally affected, with the mean seasonal hospitalization incidence rates more than twice as high than those aged 65–74 years and approximately eight times higher than adults aged 50–64 years. Mortality rates in the oldest age group (≥75 years) were also significantly elevated compared with the younger cohorts. Severity markers showed no significant differences between virus-positive and virus-negative cases or between vaccine-preventable and non-vaccine-preventable viral infections. These findings highlight the substantial respiratory health burden among adults aged ≥75 years and suggest that interventions targeting viral respiratory infections in older adults could greatly reduce the burden on the healthcare system, particularly in hospital settings.

In our study, we identified viruses in 35.2% of patients aged ≥50 years hospitalized because of respiratory illness. It is difficult to compare the rate of detection across studies because of the variations in methodology and case definitions. Our approach employed a broad yet standardized case definition for acute respiratory hospitalizations. Comparable positivity rates were reported in a US study using physician-driven testing (as part of the standard of care) [21] and in other European studies using different screening approaches [22,23]. A French study, which employed a similar case definition, found a virus detection rate (>50%) that was higher than that of our study (35.2%) [10], probably because the French study included a broad age range of adults (i.e., 18 years and older).

Importantly, consistent with previous studies, we demonstrated frequent co-circulation of several respiratory viruses during the respiratory season in Valencia, Spain, as reported in other Northern Hemisphere countries [10,11,24,25]. Nonetheless, RV/EV circulation extends beyond the conventional respiratory season, with year-round presence documented in numerous studies [8]. A study examining the distribution of viral respiratory hospitalizations in and out of the respiratory season showed that RV/EV was associated with as many hospitalizations during spring/summer as during winter [26]. This suggests that RV/EV likely impacts healthcare resource utilization throughout the year, which in turn implies a possible underestimation of hospitalization incidence rates when only the respiratory season period is considered.

Among more severe clinical presentations, RV has been associated with exacerbations of chronic respiratory illnesses, such as asthma, chronic obstructive pulmonary disease, and chronic bronchiolitis [6,7,9]. Our analysis revealed comparable rates of ICU admission, mechanical ventilation use, mortality, and LOS between patients with and without detected respiratory viruses. Furthermore, we observed no significant differences in these outcomes between patients with vaccine-preventable and non-vaccine-preventable viral infections. These findings suggest that upon hospitalization, the severity of outcomes associated with RV/EV and other non-vaccine-preventable viral infections may be comparable to those associated with vaccine-preventable viruses such as influenza and RSV, particularly among those aged ≥75 years, as the number of younger patients in these categories was less robust. The severity associated with RV/EV and other respiratory virus infections has been reported in other studies [24,27], although in-hospital severity outcomes may reflect admission thresholds and host factors rather than intrinsic viral virulence. Further investigations using more robust data and adjustments for confounders are warranted. Despite variability in study designs, many respiratory viruses have been shown to cause severe disease (including pneumonia) in older adults [28]. A multisite study in the United States suggested RV as the most common single cause of community-acquired pneumonia hospitalizations, after systematically assessing for multiple pathogens including bacteria [29]. Another prospective study conducted among selected critically ill adults, wherein other causes could not fully explain the patients’ respiratory distress and clinical presentation, showed a viral positivity rate of 40.5%, with RV/EV being the second most frequently detected viruses (after influenza) [30].

Previously, RV was considered to cause only upper respiratory tract illnesses, but clinical and experimental studies have confirmed the role of RV in lower airway illnesses as well [29,31]. Observational studies assessing the etiological role of RV by comparing its prevalence in patients with respiratory tract infections with that in matched controls without respiratory symptoms to distinguish between asymptomatic carriage and the presence of agents causing disease showed strong evidence of attribution [32,33]. Similar to our study, several studies [10,34] demonstrated that RV/EV infections were most frequently associated with hospitalizations in older adults; however, these studies were not able to distinguish the contribution of each virus. It is important to note that some patients with “viral-only” pathogens identified in specimens from their upper respiratory tract may also have bacterial infection, which was not microbiologically recognized in our study, leading to a possible inflated viral attribution. Also, some detections of viral pathogens might represent a previous or resolved virus infection rather than actual disease etiology. These aspects deserve consideration while interpreting our results. The incidence of hospitalizations for those aged ≥75 years was the highest, for each season and subgroup studies. The incidence of hospitalizations in older adults might be underestimated, because older adults are less likely to be tested and diagnosed for respiratory viruses than children [35,36], as this age group may present with atypical symptoms (e.g., confusion, anorexia, dizziness, and falls) [37,38], adding to the challenges of etiological studies among these patient populations.

The landscape of respiratory virus prevention has expanded significantly in recent years. Annual influenza vaccination has been a longstanding recommendation and licensed SARS-CoV-2 and RSV vaccines are now available for adults [5,13], both vaccines were not available at the time of this study. In Spain, the Ministry of Health recommends influenza vaccination for individuals aged ≥65 years, though some regions such as Catalonia have lowered the threshold to 60 years. Despite these recommendations, the recent influenza vaccination coverage in Spain has reached about 65% [39], falling short of the 75% target for this age group [40]. Influenza vaccination remains the most effective preventive measure to avoid influenza disease and related complications [41]. In a recent review article, influenza vaccine effectiveness among various observational studies ranged from 7.2% to 89.8% against laboratory-confirmed influenza [42]. Despite the wide range in vaccine effectiveness, evidence suggests that vaccinated individuals with breakthrough infection (influenza infection among vaccinated people) are less likely to develop severe complications and die from influenza [43,44]. The RSV vaccines are now recommended for adults aged ≥75 years. Early data from the 2023–24 season in the United States indicate a 75% vaccine effectiveness against RSV-associated hospitalizations in this age group [45]. Healthcare providers and public health officials should strongly encourage the uptake of these respiratory virus vaccinations among older adults. This approach represents a crucial public health strategy to reduce the burden of influenza, RSV, and COVID-19, offering the best individual-level protection against severe disease outcomes.

The concept of a comprehensive “respiratory vaccine” to protect vulnerable populations is gaining traction [46,47], and ongoing research includes the development of an HMPV vaccine for older adults [48]. Given the multiple viruses contributing to healthcare utilization during the typical winter respiratory season, a vaccine offering broad protection against multiple pathogens would be invaluable for both individuals and society. While age indications and target populations for these vaccines may vary, it is crucial to consider the impact of other respiratory viruses, especially RV, on hospitalizations both during and outside the respiratory season. This would be particularly important when considering the duration of protection expected from a multi-pathogen vaccine including RV. Furthermore, an RV vaccine could be especially beneficial for patients with chronic obstructive pulmonary disease or asthma conditions often triggered or exacerbated by RV infections [6,7,9]. However, the high molecular diversity of RV and limited data on the contribution of different species and subtypes to clinical presentations and their relevance for specific risk groups pose considerable obstacles [49]. Overcoming these challenges will require continued research and innovative approaches in vaccine development.

Our study has limitations. Our data might not capture hospitalizations associated with virus infections that presented without respiratory signs and symptoms (e.g., cardiac hospitalizations triggered by viral infections), which might be disproportionally more represented among older patients because of the prevalence of chronic medical conditions. Patients directly admitted to the ICU might have been missed because their consent could not be obtained. A year-round surveillance could have led to a better ascertainment of these virus-related hospitalizations because RV/EV, adenovirus, bocavirus, and HMPV can be detected outside the respiratory season [8]. Our multiplex RT-PCR assay was enhanced to detect RV, but we expected that some EV may have been misclassified as RV. The differentiation of the two groups of viruses would need to be done by sequencing to fully understand the contribution of RV to hospitalizations during the respiratory season. Nonetheless, a study differentiating RV and EV has reported that ≥60% of RV/EV positives are confirmed as RV after further viral sequencing and characterization [14], which should be especially true in the fall months when RV circulation peaks [15]. Further work differentiating RV from EV by sequencing and speciation are warranted to better understand related clinical presentations and outcomes in this population. Finally, our results may not be generalizable as healthcare utilization and admission criteria may differ in other geographic settings.

## Conclusions

The impact of respiratory viruses on hospitalizations among older adults warrants careful consideration while designing interventions to alleviate the burden on the healthcare system, particularly for those aged ≥75 years. Enhanced diagnostic capabilities would enable effective infection-control measures and the development of informed next-generation preventive strategies, including vaccines and therapies. Such a comprehensive approach would not only improve individual patient outcomes but also strengthen the resilience of the healthcare system during respiratory virus seasons.

## Supporting information

S1 Table Demographic characteristics of patients aged ≥50 years hospitalized because of respiratory illness and detected virus, by study year in Valencia, Spain during 2014–20. (DOCX)

S2 Table Mean seasonal hospitalization rates per 100,000 individuals aged ≥50 years, by age group and detection of respiratory viruses during respiratory season in Valencia, Spain during 2014–20. (DOCX)
