# Supplementary material for: Rhinovirus/enterovirus contribution to respiratory-associated hospitalizations in adults during respiratory seasons in Spain: A 6-year prospective study
Source: PLoS One. 2026 Apr 20;21(4):e0347659. doi: 10.1371/journal.pone.0347659 (PMC13095025; doi:10.1371/journal.pone.0347659)
Supplement: S1 Table — (DOCX) [file pone.0347659.s001.docx]

**S1 Table. Demographic characteristics of patients aged ≥50 years hospitalized because of respiratory illness and detected virus, by study year in Valencia, Spain during 2014–20**

| Seasons^g^ | | | | | | | | | | | | | | |
| --- | --- | --- | --- | --- | --- | --- | --- | --- | --- | --- | --- | --- | --- | --- |
|  | **2014–15 (n = 3,145)** | | **2015–16 (n = 1,566)** | | **2016–17 (n = 1,415)** | | **2017–18 (n = 1,690)** | | **2018–19 (n = 1,807)** | | **2019–20  (n = 1,052)** | | **All (N=10,675)** | |
|  | **n** | **%** | **n** | **%** | **n** | **%** | **n** | **%** | **n** | **%** | **n** | **%** | **n** | **%** |
| Age (years) | | | | | | | | | | | | | | |
| 50 to 64 | 453 | 14.4 | 278 | 17.8 | 231 | 16.3 | 311 | 18.4 | 308 | 17.0 | 207 | 19.7 | 1,788 | 16.7 |
| 65 to 74 | 696 | 22.1 | 359 | 22.9 | 338 | 23.9 | 399 | 23.6 | 389 | 21.5 | 253 | 24.0 | 2,434 | 22.8 |
| ≥75 | 1,996 | 63.5 | 929 | 59.3 | 846 | 59.8 | 980 | 58.0 | 1,110 | 61.4 | 592 | 56.3 | 6,453 | 60.4 |
| Sex | | | | | | | | | | | | | | |
| Male | 1,729 | 55.0 | 830 | 53.0 | 741 | 52.4 | 888 | 52.5 | 949 | 52.5 | 567 | 53.9 | 5,704 | 53.4 |
| Female | 1,416 | 45.0 | 736 | 47.0 | 674 | 47.6 | 802 | 47.5 | 858 | 47.5 | 485 | 46.1 | 4,971 | 46.6 |
| Ratio M/F | 1.2 | - | 1.1 | - | 1.1 | - | 1.1 | - | 1.1 | - | 1.2 | - | 1.1 | - |
| Smoking status | | | | | | | | | | | | | | |
| Never | 1,508 | 47.9 | 704 | 45.0 | 725 | 51.2 | 725 | 42.9 | 906 | 50.1 | 447 | 42.5 | 5,015 | 47.0 |
| Current | 328 | 10.4 | 245 | 15.6 | 175 | 12.4 | 261 | 15.4 | 240 | 13.3 | 178 | 16.9 | 1,427 | 13.4 |
| Former | 1,309 | 41.6 | 617 | 39.4 | 515 | 36.4 | 703 | 41.6 | 661 | 36.6 | 423 | 40.2 | 4,228 | 39.6 |
| BMI^a^ | | | | | | | | | | | | | | |
| Underweight | 48 | 1.5 | 39 | 2.5 | 31 | 2.2 | 30 | 1.8 | 50 | 2.8 | 18 | 1.7 | 216 | 2.0 |
| Normal | 947 | 30.1 | 492 | 31.4 | 442 | 31.2 | 608 | 36.0 | 559 | 30.9 | 344 | 32.7 | 3,392 | 31.8 |
| Overweight | 1,215 | 38.6 | 577 | 36.8 | 483 | 34.1 | 617 | 36.5 | 679 | 37.6 | 385 | 36.6 | 3,956 | 37.1 |
| Obese | 830 | 26.4 | 417 | 26.6 | 417 | 29.5 | 396 | 23.4 | 475 | 26.3 | 274 | 26.0 | 2,809 | 26.3 |
| Morbid obese | 105 | 3.3 | 41 | 2.6 | 42 | 3.0 | 39 | 2.3 | 44 | 2.4 | 31 | 2.9 | 302 | 2.8 |
| Functional dependency^b^ | | | | | | | | | | | | | | |
| Total | 233 | 7.4 | 144 | 9.2 | 105 | 7.4 | 118 | 7.0 | 216 | 12.0 | 56 | 5.3 | 872 | 8.2 |
| Severe | 299 | 9.5 | 183 | 11.7 | 131 | 9.3 | 173 | 10.2 | 145 | 8.0 | 110 | 10.5 | 1,041 | 9.8 |
| Moderate | 441 | 14.0 | 182 | 11.6 | 203 | 14.3 | 290 | 17.2 | 269 | 14.9 | 174 | 16.5 | 1,559 | 14.6 |
| Mild | 100 | 3.2 | 59 | 3.8 | 55 | 3.9 | 76 | 4.5 | 40 | 2.2 | 52 | 4.9 | 382 | 3.6 |
| Minimal | 1,621 | 51.5 | 720 | 46.0 | 690 | 48.8 | 722 | 42.7 | 829 | 45.9 | 453 | 43.1 | 5,035 | 47.2 |
| Missing | 451 | 14.3 | 278 | 17.8 | 231 | 16.3 | 311 | 18.4 | 308 | 17.0 | 207 | 19.7 | 1,786 | 16.7 |
| Number of comorbidities at admission | | | | | | | | | | | | | | |
| None | 313 | 10.0 | 186 | 11.9 | 184 | 13.0 | 146 | 8.6 | 164 | 9.1 | 113 | 10.7 | 1,106 | 10.4 |
| At least one | 2,832 | 90.0 | 1,380 | 88.1 | 1,231 | 87.0 | 1,544 | 91.4 | 1,643 | 90.9 | 939 | 89.3 | 9,569 | 89.6 |
| Two or more | 1,986 | 63.1 | 942 | 60.2 | 826 | 58.4 | 1,148 | 67.9 | 1,173 | 64.9 | 675 | 64.2 | 6,750 | 63.2 |
| Comorbidity^c^ | | | | | | | | | | | | | | |
| Heart disease | 1,577 | 50.1 | 835 | 53.3 | 592 | 41.8 | 931 | 55.1 | 934 | 51.7 | 560 | 53.2 | 5,429 | 50.9 |
| Cerebrovascular disease | 198 | 6.3 | 100 | 6.4 | 108 | 7.6 | 115 | 6.8 | 89 | 4.9 | 70 | 6.7 | 680 | 6.4 |
| Peripheral arteriopathy | 248 | 7.9 | 42 | 2.7 | 27 | 1.9 | 45 | 2.7 | 57 | 3.2 | 54 | 5.1 | 473 | 4.4 |
| Asthma | 270 | 8.6 | 125 | 8.0 | 122 | 8.6 | 144 | 8.5 | 175 | 9.7 | 88 | 8.4 | 924 | 8.7 |
| Lung disease | 1,233 | 39.2 | 539 | 34.4 | 435 | 30.7 | 604 | 35.7 | 646 | 35.7 | 381 | 36.2 | 3,838 | 36.0 |
| Diabetes | 1,016 | 32.3 | 518 | 33.1 | 491 | 34.7 | 601 | 35.6 | 577 | 31.9 | 349 | 33.2 | 3,552 | 33.3 |
| Endocrine system disease other than diabetes | 282 | 9.0 | 108 | 6.9 | 164 | 11.6 | 412 | 24.4 | 180 | 10.0 | 124 | 11.8 | 1,270 | 11.9 |
| Anemia | 393 | 12.5 | 178 | 11.4 | 131 | 9.3 | 160 | 9.5 | 201 | 11.1 | 88 | 8.4 | 1,151 | 10.8 |
| Chronic liver disease | 120 | 3.8 | 61 | 3.9 | 57 | 4.0 | 70 | 4.1 | 90 | 5.0 | 51 | 4.8 | 449 | 4.2 |
| Chronic renal disease | 562 | 17.9 | 243 | 15.5 | 263 | 18.6 | 274 | 16.2 | 334 | 18.5 | 183 | 17.4 | 1,859 | 17.4 |
| Chronic autoimmune disease^d^ | 165 | 5.2 | 32 | 2.0 | 59 | 4.2 | 91 | 5.4 | 111 | 6.1 | 46 | 4.4 | 504 | 4.7 |
| Neurological/Neuromuscular diseases^e^ | 307 | 9.8 | 121 | 7.7 | 149 | 10.5 | 224 | 13.3 | 279 | 15.4 | 105 | 10.0 | 1,185 | 11.1 |
| Neoplastic disease | 225 | 7.2 | 144 | 9.2 | 136 | 9.6 | 163 | 9.6 | 235 | 13.0 | 112 | 10.6 | 1,015 | 9.5 |
| Virus detected^f^ | | | | | | | | | | | | | | |
| No virus detected | 2,031 | 64.6 | 1,085 | 69.3 | 941 | 66.5 | 906 | 53.6 | 1,152 | 63.8 | 805 | 76.5 | 6,920 | 64.8 |
| Single virus detected | 1,114 | 35.4 | 481 | 30.7 | 474 | 33.5 | 784 | 46.4 | 655 | 36.2 | 247 | 23.5 | 3,755 | 35.2 |
| Vaccine-preventable virus detected | 724 | 23.0 | 256 | 16.3 | 309 | 21.8 | 570 | 33.7 | 385 | 21.3 | 117 | 11.1 | 2,361 | 22.1 |
| RV/EV detected | 210 | 6.7 | 106 | 6.8 | 102 | 7.2 | 119 | 7.0 | 189 | 10.5 | 89 | 8.5 | 815 | 7.6 |
| Other non-vaccine-preventable virus detected | 180 | 5.7 | 119 | 7.6 | 63 | 4.5 | 95 | 5.6 | 81 | 4.5 | 41 | 3.9 | 579 | 5.4 |
| ^a^The BMI categories were defined by underweight (<18.5), normal (18.5 to <25), overweight (25 to <30), obese (30 to <40), and morbid obese (≥40).  ^b^The Barthel Index data were available for only patients aged ≥65 years.  ^c^The rows did not add up to 100% as comorbidities were not mutually exclusive.  ^d^Includes acquired or hereditary immunodeficiencies and chronic autoimmune disease (e.g., lupus or rheumatoid arthritis  ^e^Includes neuromuscular or neurodegenerative disease and senile dementia or Alzheimer’s disease.  ^f^Vaccine-preventable viruses included influenza viruses and RSV; non-vaccine-preventable viruses included HMPVs, parainfluenza viruses, adenovirus, human seasonal coronavirus, and bocavirus.  ^g^Ten hospitals contributed to the season 2014–15, whereas four hospitals participated in other seasons.  BMI, body mass index; F, female; HMPV, human metapneumovirus; M, male; respiratory syncytial virus. | | | | | | | | | | | | | | |
