# Supplementary material for: Rhinovirus/enterovirus contribution to respiratory-associated hospitalizations in adults during respiratory seasons in Spain: A 6-year prospective study
Source: PLoS One. 2026 Apr 20;21(4):e0347659. doi: 10.1371/journal.pone.0347659 (PMC13095025; doi:10.1371/journal.pone.0347659)
Supplement: S2 Table — (DOCX) [file pone.0347659.s002.docx]

**S2 Table.** **Mean seasonal hospitalization rates per 100,000 individuals aged ≥50 years, by age group and detection of respiratory viruses during respiratory season in Valencia, Spain during 2014–20**

|  | 50 to 64 years | 65 to 74 years | ≥75 years | All |
| --- | --- | --- | --- | --- |
|  | **Mean (min; max)** | **Mean**  **(min; max)** | **Mean (min; max)** | **Mean**  **(min; max)** |
| All acute respiratory-associated hospitalizations | 118.1  (88.4; 144.6) | 328.9  (228.1; 381.9) | 925.9  (570.4; 1,095.7) | 357.5  (234.4; 410.4) |
| Hospitalizations associated with a single respiratory virus | 38.2  (23.1; 56.7) | 116.7  (56.8; 168.5) | 332.6  (125.3; 507.9) | 125.8  (55; 188.8) |
| Hospitalizations associated with vaccine-preventable viruses | 23.1 (12; 38.6) | 73,2 (24.3; 130.2) | 209.7 (59.7; 366.8) | 78.8 (26.1; 137.3) |
| Hospitalizations associated with RV/EV | 9.9 (7.6; 13.6) | 25 (18.2; 35.3) | 71.1 (43.4; 117.5) | 27.8 (19.8; 42.9) |
| Hospitalizations associated with other non-vaccine-preventable viruses | 5.3 (3; 8.1) | 18.5 (9.9; 33) | 51.8 (22.2; 73.1) | 19.3 (9.1; 29.5) |

Min, minimum; Max, maximum; RSV, RV/EV, Rhinovirus/Enterovirus
